# Supplementary material for: Looking upstream: enhancers of child nutritional status in post-flood rural settings
Source: PeerJ. 2016 Mar 1;4:e1741. doi: 10.7717/peerj.1741 (PMC4782687; doi:10.7717/peerj.1741)
Supplement: Supplemental Information 1 [file peerj-04-1741-s001.docx]

**Supplemental Information**

**Table S1: Univariate logistic regression models for maternal and paternal education, and other risk factors related to stunting in flooded and non-flooded children populations aged 6 to 59 months in rural Odisha, India.**

|  |  | Flooded (*n* = 299) | | Non-flooded (*n* = 385) | |
| --- | --- | --- | --- | --- | --- |
|  |  | PR (95% CI) | p-value | PR (95% CI) | p-value |
| Mother age at marriage (years) | | 0.920 (0.853, 0.991) | 0.03 | 0.954 (0.880, 1.034) | 0.26 |
| Mother age at first delivery (years) | | 0.928 (0.864, 0.998) | 0.05 | 0.951 (0.885, 1.021) | 0.17 |
| Mother age at birth of selected child (years) | | 0.971 (0.917, 1.028) | 0.31 | 0.983 (0.940, 1.027) | 0.44 |
| Maternal education | | .. | .. | .. | .. |
|  | No schooling | 1 | .. | 1 | .. |
|  | Primary school | 0.852 (0.481, 1.510) | 0.58 | 1.096 (0.601, 1.998) | 0.765 |
|  | Middle school | 0.436 (0.241, 0.789) | 0.01 | 0.794 (0.424, 1.486) | 0.471 |
|  | High school | 0.558 (0.293, 1.062) | 0.08 | 0.460 (0.228, 0.928) | 0.031 |
|  | College or more | 0.334 (0.131, 0.851) | 0.02 | 0.581 (0.213, 1.584) | 0.290 |
| Father age at birth of selected child (years) | | 0.964 (0.917, 1.013) | 0.15 | 0.972 (0.938, 1.008) | 0.13 |
| Paternal education | | .. | .. | .. | .. |
|  | No schooling | 1 | .. | 1 | .. |
|  | Primary school | 0.592 (0.363, 0.964) | 0.04 | 0.469 (0.261, 0.843) | 0.012 |
|  | Middle school | 0.277 (0.170, 0.453) | <0.0001 | 0.431 (0.256, 0.724) | 0.002 |
|  | High school | 0.330 (0.207, 0.524) | <0.0001 | 0.455 (0.275, 0.754) | 0.002 |
|  | College or more | 0.314 (0.168, 0.590) | <0.0001 | 0.295 (0.140, 0.618) | 0.001 |
| Child sex (girl vs boy) | | 1.034 (0.683, 1.564) | 0.88 | 0.827 (0.576, 1.188) | 0.31 |
| Child birthweight (per 100 g) | | 0.934 (0.896, 0.974) | 0.00 | 0.947 (0.904, 0.993) | 0.03 |
| Child age (months) | | 1.016 (1.003, 1.029) | 0.01 | 1.015 (1.004, 1.026) | 0.01 |
| Number of children (< 5 years) eating from same kitchen | | 1.199 (0.946, 1.521) | 0.14 | 0.986 (0.810, 1.200) | 0.89 |
| Means of livelihood (non-agricultural vs agricultural) | | 1.065 (0.686, 1.655) | 0.79 | 1.049 (0.692, 1.588) | 0.82 |
| Religion (hindu vs muslim) | | 0.918 (0.583, 1.446) | 0.71 | 1.806 (0.755, 4.321) | 0.19 |
| Caste | | .. | .. | .. | .. |
|  | General | 1 | .. | 1 | .. |
|  | Other backward | 1.792 (0.908, 3.537) | 0.09 | 1.043 (0.607, 1.793) | 0.88 |
|  | Scheduled caste | 2.237 (1.128, 4.436) | 0.02 | 1.181 (0.660, 2.115) | 0.58 |
|  | No caste | 1.836 (0.900, 3.746) | 0.10 | 0.597 (0.224, 1.593) | 0.30 |
| Land owned (hectare) | | 0.614 (0.366, 1.031) | 0.07 | 0.880 (0.529, 1.465) | 0.62 |
| Per head annual income (per 1,000 rupees) | | 0.941 (0.894, 0.991) | 0.02 | 0.997 (0.972, 1.023) | 0.83 |
| Livestock owned (none vs any) | | 1.358 (0.901, 2.047) | 0.15 | 0.961 (0.666, 1.388) | 0.83 |
| Number of individuals eating from same kitchen | | .. | .. | .. | .. |
|  | 2-4 | 1 | .. | 1 | .. |
|  | >4 | 1.661 (0.839, 3.291) | 0.15 | 0.781 (0.539, 1.133) | 0.19 |

**Table S2: Univariate logistic regression models for maternal and paternal education, and other risk factors related to wasting in flooded and non-flooded children populations aged 6 to 59 months in rural Odisha, India.**

|  |  | Flooded (*n* = 299) | | Non-flooded (*n* = 385) | |
| --- | --- | --- | --- | --- | --- |
|  |  | PR (95% CI) | p-value | PR (95% CI) | p-value |
| Mother age at marriage (years) | | 0.960 (0.915, 1.006) | 0.09 | 0.973 (0.871, 1.086) | 0.62 |
| Mother age at first delivery (years) | | 0.963 (0.920, 1.010) | 0.12 | 0.964 (0.873, 1.065) | 0.47 |
| Mother age at birth of selected child (years) | | 0.964 (0.931, 0.999) | 0.04 | 0.983 (0.932, 1.037) | 0.53 |
| Maternal education | | .. | .. | .. | .. |
|  | No schooling | 1 | .. | 1 | .. |
|  | Primary school | 0.789 (0.450, 1.383) | 0.41 | 0.994 (0.451, 2.190) | 0.988 |
|  | Middle school | 1.084 (0.669, 1.758) | 0.74 | 0.418 (0.166, 1.052) | 0.065 |
|  | High school | 0.899 (0.536, 1.508) | 0.69 | 0.415 (0.176, 0.977) | 0.045 |
|  | College or more | 0.872 (0.483, 1.574) | 0.65 | 0.866 (0.310, 2.429) | 0.785 |
| Father age at birth of selected child (years) | | 0.993 (0.962, 1.024) | 0.65 | 0.964 (0.920, 1.012) | 0.14 |
| Paternal education | | .. | .. | .. | .. |
|  | No schooling | 1 | .. | 1 | .. |
|  | Primary school | 0.813 (0.458, 1.444) | 0.48 | 0.310 (0.125, 0.750) | 0.010 |
|  | Middle school | 0.623 (0.366, 1.060) | 0.08 | 0.318 (0.140, 0.725) | 0.007 |
|  | High school | 0.786 (0.470, 1.313) | 0.36 | 0.340 (0.160, 0.725) | 0.005 |
|  | College or more | 0.587 (0.323, 1.066) | 0.08 | 0.451 (0.198, 1.030) | 0.060 |
| Child sex (girl vs boy) | | 0.978 (0.756, 1.265) | 0.87 | 0.736 (0.434, 1.248) | 0.26 |
| Child birthweight (per 100 g) | | 0.991 (0.957, 1.027) | 0.62 | 0.966 (0.898, 1.040) | 0.36 |
| Child age (months) | | 1.000 (0.992, 1.009) | 0.96 | 0.976 (0.957, 0.996) | 0.02 |
| Number of children (< 5 years) eating from same kitchen | | 1.103 (0.950, 1.280) | 0.20 | 0.972 (0.756, 1.251) | 0.83 |
| Means of livelihood (non-agricultural vs agricultural) | | 0.681 (0.529, 0.877) | 0.003 | 0.750 (0.442, 1.273) | 0.29 |
| Religion (hindu vs muslim) | | 0.919 (0.692, 1.222) | 0.56 | 1.126 (0.449, 2.826) | 0.80 |
| Caste | | .. | .. | .. | .. |
|  | General | 1 | .. | 1 | .. |
|  | Other backward | 1.406 (1.000, 1.975) | 0.051 | 0.736 (0.371, 1.460) | 0.38 |
|  | Scheduled caste | 1.117 (0.743, 1.680) | 0.596 | 1.054 (0.500, 2.222) | 0.89 |
|  | No caste | 1.240 (0.838, 1.835) | 0.282 | 0.798 (0.278, 2.288) | 0.67 |
| Land owned (hectare) | | 0.955 (0.816, 1.117) | 0.56 | 1.196 (0.809, 1.771) | 0.37 |
| Per head annual income (per 1,000 rupees) | | 0.985 (0.964, 1.008) | 0.20 | 0.998 (0.975, 1.022) | 0.90 |
| Livestock owned (none vs any) | | 1.039 (0.800, 1.349) | 0.78 | 1.118 (0.665, 1.879) | 0.68 |
| Number of individuals eating from same kitchen | | .. | .. | .. | .. |
|  | 2-4 | 1 | .. | 1 | .. |
|  | >4 | 1.154 (0.805, 1.653) | 0.44 | 1.710 (0.938, 3.116) | 0.08 |

**STROBE Statement—Checklist of items that should be included in reports of *cross-sectional studies***

**Article Title: “Looking upstream: enhancers of child nutritional status in post-flood rural settings”.**

|  | Item No | Recommendation |
| --- | --- | --- |
| **Title and abstract** | 1🗸  p1-2 | (*a*) Indicate the study’s design with a commonly used term in the title or the abstract (NOT DONE, not required by the Journal) |
|  |  | (*b*) Provide in the abstract an informative and balanced summary of what was done and what was found |
| Introduction | | |
| Background/rationale | 2🗸  p3-4 | Explain the scientific background and rationale for the investigation being reported |
| Objectives | 3🗸  p4 | State specific objectives, including any prespecified hypotheses |
| Methods | | |
| Study design | 4🗸  p4-5 | Present key elements of study design early in the paper |
| Setting | 5🗸  p6 | Describe the setting, locations, and relevant dates, including periods of recruitment, exposure, follow-up, and data collection |
| Participants | 6🗸  p4-6 | (*a*) Give the eligibility criteria, and the sources and methods of selection of participants |
| Variables | 7🗸  p7-9 | Clearly define all outcomes, exposures, predictors, potential confounders, and effect modifiers. Give diagnostic criteria, if applicable |
| Data sources/ measurement | 8🗸  p7 | For each variable of interest, give sources of data and details of methods of assessment (measurement). Describe comparability of assessment methods if there is more than one group |
| Bias | 9🗸  p9-10 | Describe any efforts to address potential sources of bias |
| Study size | 10🗸 | Explain how the study size was arrived at (NOT DONE as the study used existing data) |
| Quantitative variables | 11🗸  p7-8 | Explain how quantitative variables were handled in the analyses. If applicable, describe which groupings were chosen and why |
| Statistical methods | 12🗸  p9-10 | (*a*) Describe all statistical methods, including those used to control for confounding (OK) |
|  |  | (*b*) Describe any methods used to examine subgroups and interactions (OK) |
|  |  | (*c*) Explain how missing data were addressed (they were rare, no need to be addressed) |
|  |  | (*d*) If applicable, describe analytical methods taking account of sampling strategy (DONE) |
|  |  | (*e*) Describe any sensitivity analyses (small sample, NOT DONE) |
| Results | | |
| Participants | 13🗸  p10 | (a) Report numbers of individuals at each stage of study—eg numbers potentially eligible, examined for eligibility, confirmed eligible, included in the study, completing follow-up, and analysed – Fig 2 |
|  |  | (b) Give reasons for non-participation at each stage 🗸 Fig 2 |
|  |  | (c) Consider use of a flow diagram 🗸 Fig 2 |
| Descriptive data | 14🗸  p10, table 1 | (a) Give characteristics of study participants (eg demographic, clinical, social) and information on exposures and potential confounders |
|  |  | (b) Indicate number of participants with missing data for each variable of interest |
| Outcome data | 15🗸  p10, table1 | Report numbers of outcome events or summary measures |
| Main results | 16🗸  p13-17, table2-3  n/a | (*a*) Give unadjusted estimates and, if applicable, confounder-adjusted estimates and their precision (eg, 95% confidence interval). Make clear which confounders were adjusted for and why they were included 🗸 Fig. 3-4 |
|  |  | (*b*) Report category boundaries when continuous variables were categorized 🗸 |
|  |  | (*c*) If relevant, consider translating estimates of relative risk into absolute risk for a meaningful time period |
| Other analyses | 17🗸  n/a | Report other analyses done—eg analyses of subgroups and interactions, and sensitivity analyses |
| Discussion | | |
| Key results | 18🗸  p18 | Summarise key results with reference to study objectives |
| Limitations | 19🗸  P20-21 | Discuss limitations of the study, taking into account sources of potential bias or imprecision. Discuss both direction and magnitude of any potential bias |
| Interpretation | 20🗸  p20-21 | Give a cautious overall interpretation of results considering objectives, limitations, multiplicity of analyses, results from similar studies, and other relevant evidence |
| Generalisability | 21🗸  p21 | Discuss the generalisability (external validity) of the study results |
| Other information | | |
| Funding | 22, p27 | Give the source of funding and the role of the funders for the present study and, if applicable, for the original study on which the present article is based |
